# Epstein-Barr Virus (EBV)-Positive Diffuse Large B-cell Lymphoma Masquerading As Lupus Pneumonitis in a Young Woman With Systemic Lupus Erythematosus: A Diagnostic Odyssey

**DOI:** 10.7759/cureus.108460

**Published:** 2026-05-07

**Authors:** Onyekachi O Anya, Shariff Mosallaei, Nicholas Wysham

**Affiliations:** 1 Internal Medicine, Legacy Salmon Creek Medical Center, Vancouver, USA; 2 Pulmonary and Critical Care, Vancouver Clinic, Vancouver, USA

**Keywords:** diagnostic delay, diffuse large b-cell lymphoma, ebv-positive lymphoma, immunodeficiency-associated lymphoproliferative disorder, pulmonary nodules, systemic lupus erythematosus

## Abstract

Patients with systemic lupus erythematosus (SLE) are at increased risk for lymphoma compared to the general population. While pulmonary manifestations are common in SLE, diffuse micronodular patterns can resemble infection, granulomatous disease, or malignancy, making diagnosis challenging.

A 21-year-old woman with childhood-onset SLE (class IV lupus nephritis) on mycophenolate mofetil and hydroxychloroquine presented with progressive dyspnea and diffuse bilateral pulmonary micronodularity. Initial bronchoscopy and biopsy showed non-specific perivascular inflammation, negative for malignancy or infection. She initially responded to steroids with near-complete radiographic resolution. Over the next nine months, however, she developed recurrent pulmonary nodules unresponsive to increased immunosuppression. Repeat bronchoscopy revealed necrotizing granulomatous inflammation without identifiable organisms. Her condition progressed to respiratory failure, necessitating a surgical lung biopsy. Video-assisted thoracoscopic surgery (VATS) wedge resection identified Epstein-Barr virus (EBV)-positive diffuse large B-cell lymphoma arising in immune deficiency/dysregulation, with an EBV PCR of 25,500 IU/mL. Postoperatively, she developed acute respiratory failure requiring extracorporeal membrane oxygenation (ECMO) and hemophagocytic lymphohistiocytosis (HLH). She completed six cycles of rituximab, cyclophosphamide, doxorubicin, vincristine, and prednisone (R-CHOP) chemotherapy. End-of-treatment PET/CT showed persistent hypermetabolic pulmonary nodules (Deauville score 5), and she is now being evaluated for chimeric antigen receptor (CAR) T-cell therapy.

This case highlights the challenge of distinguishing lymphoproliferative disorders from autoimmune lung disease in immunosuppressed SLE patients. Initial steroid responsiveness does not rule out malignancy. Clinicians should maintain a high suspicion for lymphoma in SLE patients with atypical or refractory pulmonary symptoms, especially those on chronic immunosuppression.

## Introduction

Systemic lupus erythematosus (SLE) is a multisystem autoimmune disorder characterized by immune dysregulation and chronic inflammation. Pulmonary involvement is prevalent, affecting 50-70% of SLE patients over the course of their disease, and includes pleuritis, interstitial lung disease, pulmonary hypertension, diffuse alveolar hemorrhage, acute lupus pneumonitis, and shrinking lung syndrome [1]. Although collectively common, individual pulmonary manifestations other than pleural disease are uncommon, each occurring in fewer than 4% of patients [2]. Diffuse micronodular infiltrates are atypical for SLE and may mimic infection, granulomatous disease, or malignancy [3].

Patients with SLE have a well-established four- to seven-fold increased risk of non-Hodgkin lymphoma compared to the general population, with diffuse large B-cell lymphoma (DLBCL) being the most consistently associated subtype [4]. Whether this elevated risk is attributable to the underlying immune dysregulation, chronic antigenic stimulation, or the immunosuppressive therapies used in management is incompletely understood [4]. Chronic immunosuppression impairs T-cell surveillance of Epstein-Barr virus (EBV)-infected B cells, enabling unregulated viral proliferation and predisposing to EBV-driven lymphoproliferative disorders [5]. EBV-positive DLBCL arising in immune deficiency/dysregulation is now recognized as a distinct entity in both the WHO (2024) and International Consensus Classification (2022), analogous to post-transplant lymphoproliferative disorder (PTLD) but occurring in non-transplant settings of immunosuppression [5]. Notably, EBV-associated lymphomas may initially regress with reduction of immunosuppression, a feature that may paradoxically delay definitive diagnosis [6].

Pulmonary involvement by lymphoma is uncommon and diagnostically challenging. Primary pulmonary lymphoma accounts for less than 0.5% of all primary lung tumors, and its clinical and radiographic features frequently mimic pneumonia, granulomatous infection, or inflammatory lung disease [7]. In a retrospective series, 68% of primary pulmonary lymphoma cases were initially misdiagnosed as pneumonia, lung cancer, or tuberculosis. The time between initial and final diagnosis ranged from half a month to two years, with a median of six months [8]. The diagnostic difficulty is compounded in immunocompromised patients, where infection and malignancy may coexist or present with overlapping features [9].

We describe a young woman with childhood-onset SLE on chronic immunosuppression whose progressive pulmonary disease was initially presumed to represent lupus pneumonitis but was ultimately diagnosed as EBV-positive DLBCL following surgical lung biopsy. This case illustrates the diagnostic pitfalls of atypical pulmonary presentations in immunosuppressed SLE patients and stresses the importance of maintaining a strong index of suspicion for lymphoproliferative disorders when pulmonary disease is refractory to immunosuppressive therapy.

## Case presentation

Initial presentation

A 21-year-old woman with childhood-onset SLE presented to the emergency department with five days of fatigue, headaches, poor sleep, and mild shortness of breath. She had been diagnosed with SLE at age 13 in 2018, presenting with weight loss, joint pain, malar rash, oral ulcers, anemia, leukopenia, anti-dsDNA >300 IU/mL, antinuclear antibody (ANA) 1:2560, hypocomplementemia with low complement levels (C3: 38 mg/dL, C4: 3 mg/dL), and proteinuria (urine protein-to-creatinine ratio (UPC) 1.04). Renal biopsy confirmed WHO class IV lupus nephritis. Her SLE had been well-controlled on mycophenolate mofetil 1000 mg twice daily and hydroxychloroquine 400 mg daily, with no prednisone requirement since shortly after diagnosis.

She reported a 12-pound unintentional weight loss over four to six weeks, nausea, early satiety, and a non-productive cough. She denied fevers. Her social history included remote travel to Europe, with no exposure to tuberculosis-endemic areas, jails, or shelters. She did not use tobacco but reported tetrahydrocannabinol (THC) vaping. She appeared fatigued but was in no acute distress. Lungs were clear to auscultation bilaterally. No lymphadenopathy was appreciated. Skin examination revealed ecchymoses on the neck but was otherwise unremarkable. Laboratory findings are summarized in Table 1.

**Table 1:** Lab results WBC: white blood count; Hb: hemoglobin; HCT: hematocrit; BUN: blood urea nitrogen; K/cu mm: thousands per cubic millimeter; g/dL: grams per deciliter; mmol/L: millimoles per liter; mg/dL: milligram per deciliter

Test	Results	Reference range and units
WBC	3.14	3.50-10.80 K/cu mm
Hb	13.9	12.0-16.0 g/dL
HCT	41.7	36.0-46.0%
Platelet	249	150-400 K/cu mm
Sodium	137	136-145 mmol/L
Potassium	3.9	3.4-5.0 mmol/L
Chloride	104	97-108 mmol/L
CO_2_	24	21-32 mmol/L
BUN	10	6-20 mg/dL
Creatinine	0.57	0.60-1.10 mg/dL
Glucose	92	70-99 mg/dL
Calcium	8.9	8.6-10.2 mg/dL

Investigations

**Figure 1:**
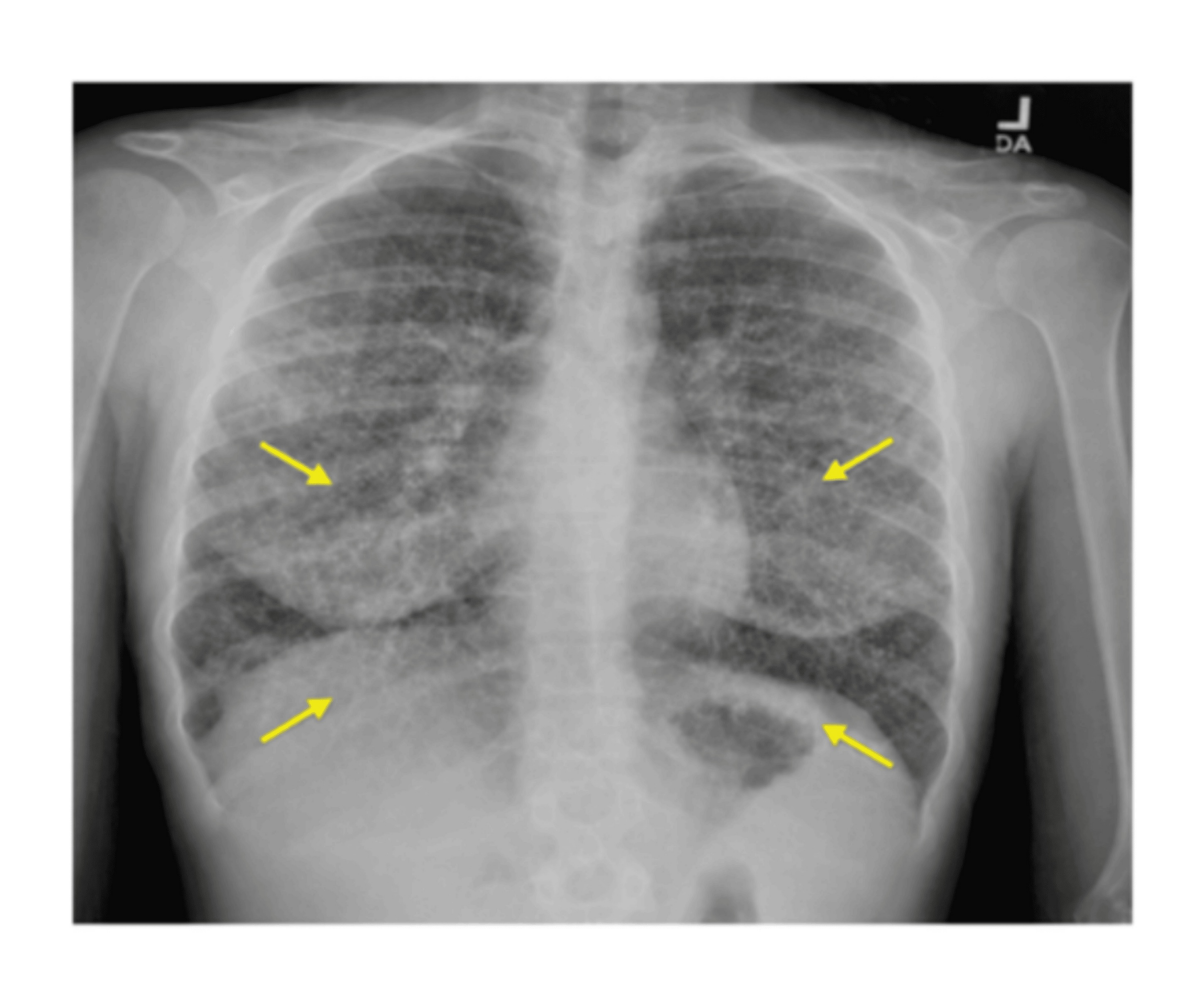
Initial chest X-ray showing bilateral micronodular opacities Frontal chest radiograph demonstrates diffuse, bilateral micronodular opacities (arrows) involving the mid to lower lung zones, more pronounced in the perihilar and peripheral regions. Cardiomediastinal silhouette and costophrenic angles are within normal limits.

**Figure 2:**
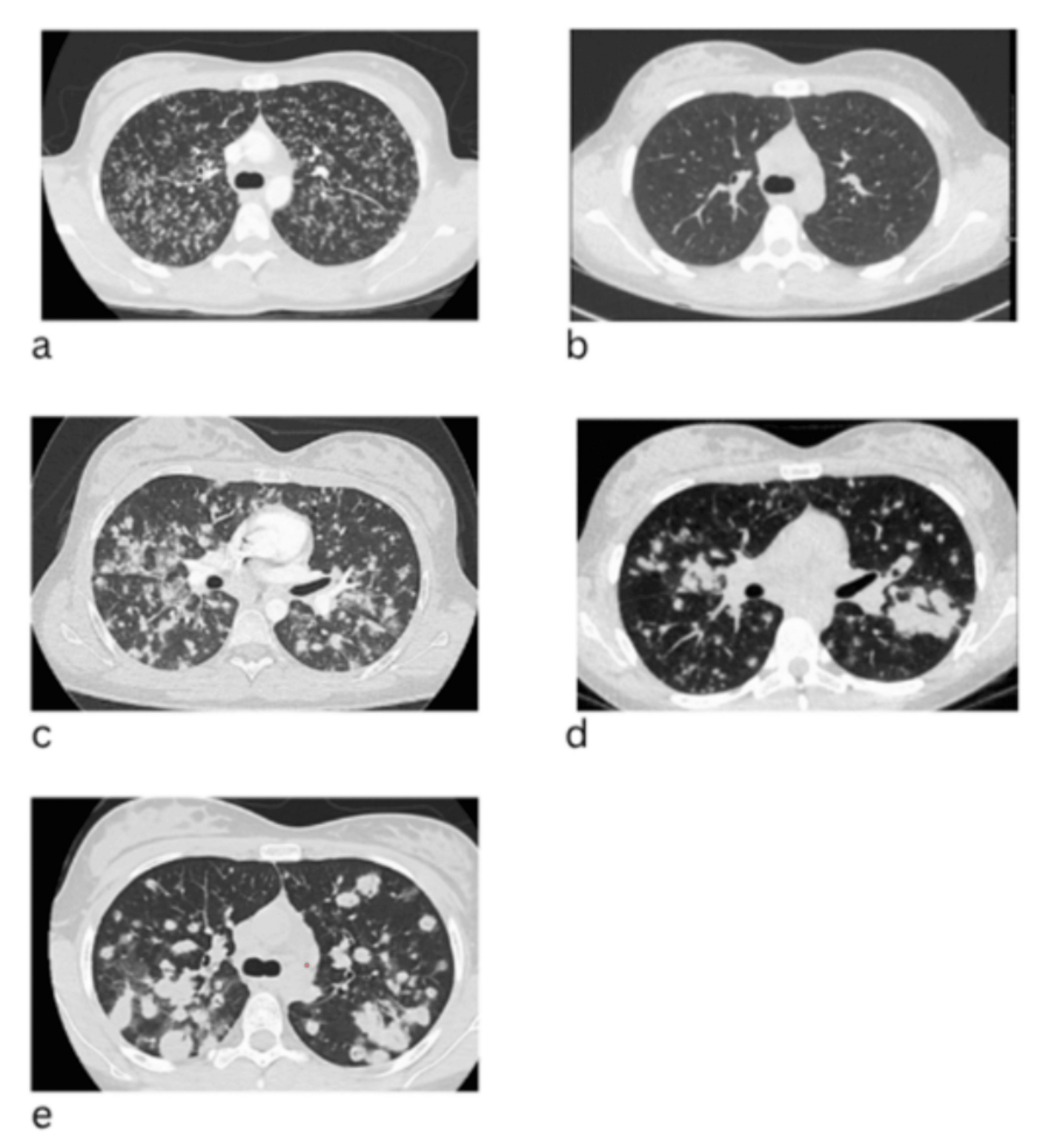
CT scan progression Image a: Reveals diffuse ground-glass and solid miliary nodules bilaterally with mild hilar adenopathy. Image b: Marked steroid response; Showed near complete resolution of the miliary micro nodularity, with only a few residual nodules. Image c: Six-month CT revealed recurrent diffuse miliary nodules that had increased in size (5-10 mm) and become more solid. Image d: CT chest three months after showed persistent, enlarging nodules. Image e: Chest CT demonstrating diffuse bilateral nodules with areas of coalescence and a 5.6-cm peripheral cyst/bulla in the right upper lobe.

Chest X-ray showed numerous bilateral micronodular opacities (Figure 1). Chest CT revealed diffuse ground-glass and solid miliary nodules bilaterally with mild hilar adenopathy (Figure 2a).

**Figure 3:**
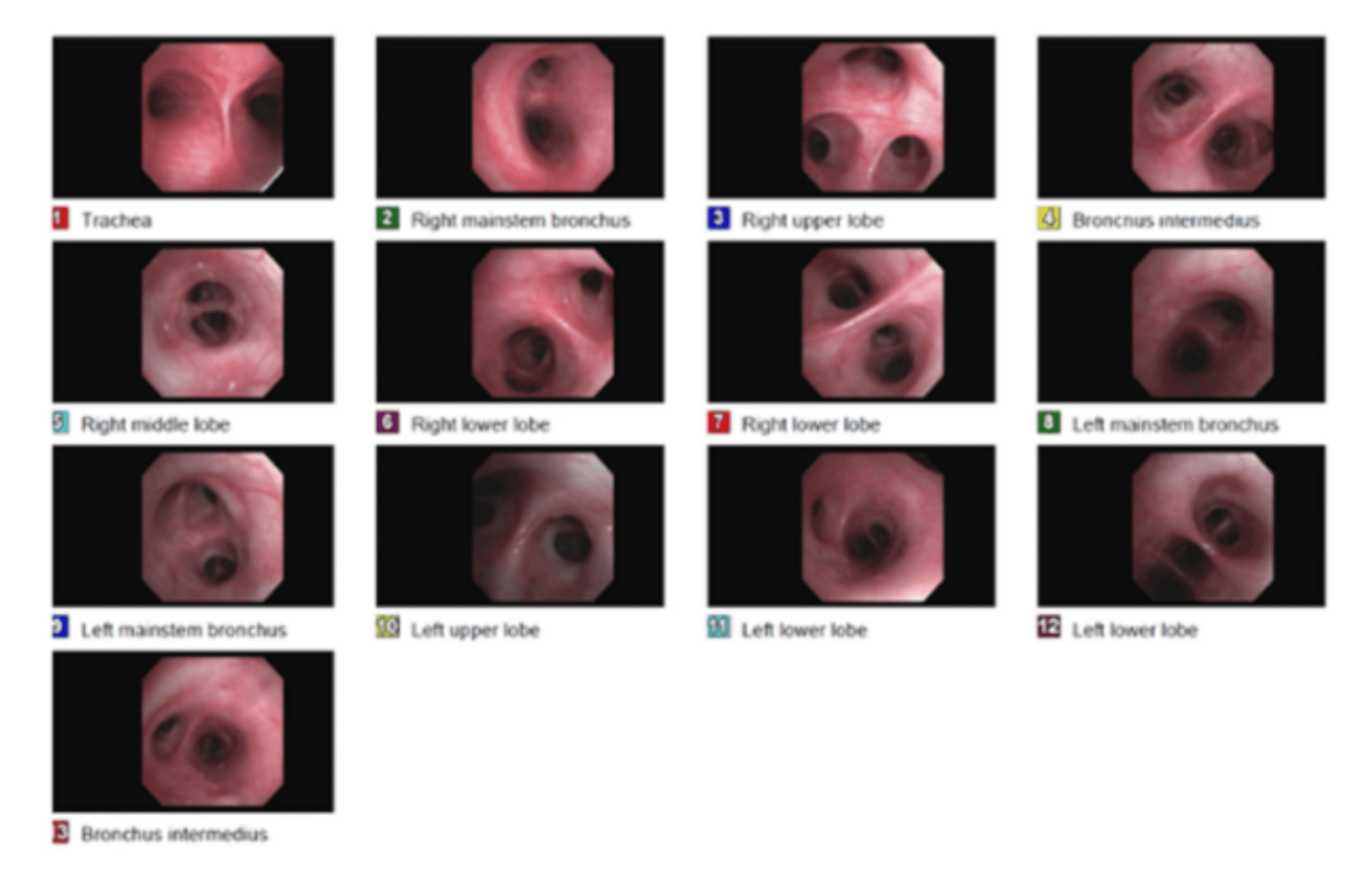
Bronchoscopy and lung findings Bronchoscopy revealed grossly normal airway mucosa with no endobronchial lesions, masses, or signs of inflammation. These normal findings underscore that EBV-positive diffuse large B-cell lymphoma cannot be excluded based on bronchoscopic appearance alone, and the diagnosis was ultimately confirmed by VATS biopsy. EBV: Epstein-Barr virus; VATS: video-assisted thoracoscopic surgery

Bronchoscopy with bronchoalveolar lavage (BAL) of the lingula and transbronchial biopsy of the right middle lobe was performed on hospital day 5 following initial infectious evaluation and pulmonology consultation (Figure 3). The procedure was complicated by a right-sided pneumothorax requiring placement of a pigtail catheter. BAL analysis demonstrated 41% lymphocytes. An extensive infectious evaluation was negative, including bacterial and fungal cultures, acid-fast bacilli cultures (with a single isolate of *Mycobacterium gordonae* interpreted as a nonpathogenic contaminant), and testing for *Pneumocystis jirovecii*, *Legionella*, cytomegalovirus (CMV), herpes simplex virus (HSV), *Histoplasma* antigen, *Coccidioides* antibody, and cryptococcal antigen.

Pathology from the transbronchial biopsy was sent to the Mayo Clinic for expert review and demonstrated an atypical mixed perivascular infiltrate with focal fibrinous acute lung injury. No evidence of lymphoproliferative disease or malignancy was identified. The antineutrophil cytoplasmic antibody (ANCA) and hypersensitivity pneumonitis panels were negative. A concurrent truncal skin rash was biopsied and revealed non-caseating granulomas.

Initial treatment and response

Given the negative infectious workup and her history of SLE, an autoimmune etiology was considered, and prednisone 20 mg daily was initiated. Vaping-associated lung injury (EVALI) was also considered, given her prior THC vaping, although she denied current use. Follow-up chest CT a month after showed near-complete resolution of the miliary micronodularity, with only a few residual nodules. This marked steroid response was interpreted as evidence for an autoimmune or inflammatory process (see Figure 2b).

Disease recurrence and progression

Six months later, she presented with recurrent dyspnea, and chest CT revealed recurrent diffuse miliary nodules that had increased in size (5-10 mm) and become more solid. Prednisone was restarted at 10 mg daily, and mycophenolate was increased to 1,250 mg twice daily (see Figure 2c).

Despite escalating immunosuppression, a CT chest three months after showed persistent, enlarging nodules (Figures 2d-2e). Prednisone was increased to 40 mg daily, and mycophenolate to 1500 mg twice daily. Repeat bronchoscopy with endobronchial ultrasound (EBUS)-guided biopsy and cryobiopsy was performed. Pathology from right upper lobe specimens and station 7 lymph node revealed necrotizing granulomatous inflammation. Stains for acid-fast bacilli and fungi were negative. BAL flow cytometry showed no evidence of lymphoproliferative disorder. BAL *Aspergillus* galactomannan was mildly elevated (0.57, >0.5 positive), though fungitell was negative.

She was admitted post-procedure with hypoxia requiring supplemental oxygen. Treatment included methylprednisolone 125 mg IV every six hours, followed by discharge on prednisone 60 mg daily and atovaquone for *P. jirovecii* pneumonia (PCP) prophylaxis.

Clinical deterioration and definitive diagnosis

Three weeks after discharge, she presented with fevers up to 101.6°F and worsening dyspnea; chest X-ray showed extensive confluent pulmonary nodules and she was treated empirically for pneumonia with Augmentin and azithromycin, but was readmitted one week later after discharge with recurrent fevers and worsening respiratory failure, with chest CT demonstrating diffuse bilateral nodules with areas of coalescence and a 5.6-cm peripheral cyst/bulla in the right upper lobe, interpreted as most consistent with airway-related granulomatous disease likely infectious in origin; she was subsequently transferred to a tertiary care center several days later for surgical lung biopsy, and underwent video-assisted thoracoscopic surgery (VATS) right upper lobe wedge resection the following day.

Final pathology showed DLBCL arising in immune deficiency/dysregulation (PTLD-like). The tumor showed an atypical polymorphous infiltrate of variably sized lymphocytes, including numerous large, atypical B cells. Immunophenotyping showed positivity for CD20, MUM1, CD79a, and BCL6, with lambda light-chain restriction and patchy CD30 expression. EBV-encoded RNA (EBER) was positive. The Ki-67 proliferation index was 50%. EBV PCR (plasma) showed 25,500 IU/mL.

Critical illness and treatment

Her VATS was complicated by acute respiratory failure requiring continued intubation and initiation of venovenous ECMO several days later. Hydroxychloroquine and mycophenolate were discontinued. Her course was further complicated by the development of hemophagocytic lymphohistiocytosis (HLH), with CRP of 141.9, ferritin of 4,127, soluble IL-2 receptor of 3,440.7, and triglycerides of 566; she was treated with anakinra 100 mg twice daily for seven days, followed by 100 mg daily for four days. She subsequently received her first cycle of rituximab, cyclophosphamide, doxorubicin, vincristine, and prednisone (R-CHOP) several days later and was successfully decannulated from ECMO after 12 days of support. Her hospitalization was further complicated by bilateral lower-extremity deep vein thromboses involving the femoral, popliteal, and calf veins, for which apixaban 5 mg twice daily was initiated. She was ultimately discharged after a 22-day hospitalization.

Subsequent treatment course

She completed six cycles of R-CHOP. Interim PET/CT after three cycles showed improved but persistent disease with residual bilateral pulmonary nodules, including a right upper lobe nodule measuring 21 mm with SUVmax 16.8.

Current status and follow-up

End-of-treatment PET/CT demonstrated persistent hypermetabolic pulmonary nodules concerning for residual disease, including a right upper lobe nodule measuring 15 mm (decreased from 21 mm) with SUVmax 14.1 (previously 16.8), with a reference liver SUVmax of 2.9 and a Deauville score of 5, consistent with persistent metabolically active disease. At her most recent clinic visit, she reported feeling well with an Eastern Cooperative Oncology Group (ECOG) performance status of 0. Given the Deauville 5 response indicating refractory disease, she was referred for chimeric antigen receptor (CAR) T-cell therapy evaluation, with ClonoSeq testing planned to assess minimal residual disease and CT-guided biopsy of the residual pulmonary nodule if results are inconclusive. The timeline of key events in this case is illustrated in Table 2.

**Table 2:** Timeline of key events CT: computed tomography; BAL: bronchoalveolar lavage; EBV: Epstein-Barr virus; DLBCL: diffuse large B-cell lymphoma; ECMO: extracorporeal membrane oxygenation; HLH: hemophagocytic lymphohistiocytosis; R-CHOP: rituximab, cyclophosphamide, doxorubicin, vincristine, and prednisone; PET: positron emission tomography; ECOG: Eastern Cooperative Oncology Group; VATS: video-assisted thoracoscopic surgery

Time	Clinical event
January 2025	Initial presentation with dyspnea.
January 2025	Diffuse micronodules on CT.
January 2025	BAL negative.
February 2025	Response to steroid.
June 2025	Recurrence of nodules.
September 2025	Necrotizing granulomas on bronchoscopy.
October 2025	VATS biopsy → EBV+ DLBCL.
October 2025	ECMO + HLH.
October 2025	R-CHOP initiated.
December 2025	Partial response PET.
March 2026	PET/CT demonstrated persistent hypermetabolic pulmonary nodules concerning for residual disease. Deauville score of 5 and ECOG of 0.

## Discussion

Patients with SLE have a four- to seven-fold increased risk of non-Hodgkin lymphoma compared to the general population, with DLBCL being the most common subtype [10]. The pathogenesis is multifactorial: chronic immune activation drives B-cell proliferation, while impaired T-cell surveillance, from both the underlying disease and iatrogenic immunosuppression, permits survival of malignant clones [10]. Disease activity itself appears to be a stronger predictor of lymphoma risk than any individual immunosuppressive agent, though cumulative corticosteroid exposure has been independently associated with increased risk [11]. EBV plays a central role in lymphomagenesis in immunosuppressed patients. EBV establishes a lifelong latent infection in B lymphocytes, which are normally held in check by cytotoxic T-cell surveillance [12]. When this surveillance is impaired, unregulated EBV-driven B-cell proliferation can lead to aggressive lymphomas [5]. EBV-positive DLBCL arising in immune deficiency/dysregulation is now recognized as a distinct entity in the WHO 2024 classification, analogous to PTLD but occurring in non-transplant settings [5].

Our patient's EBV viral load of 25,500 IU/mL at diagnosis is consistent with this pathophysiology. Pulmonary lymphoma constitutes a major diagnostic challenge, as its radiographic findings of masses, consolidations, or diffuse nodular infiltrates frequently mimic infectious processes [8]. In one retrospective series, 68% of primary pulmonary lymphoma cases were initially misdiagnosed as pneumonia, tuberculosis, or lung cancer, with a median time to correct diagnosis of six months [8]. In our patient, the diffuse miliary pattern initially raised concern for granulomatous infection, and the radiologist's interpretation of findings most consistent with infectious pathology illustrates how pulmonary lymphoma can be indistinguishable from infection on imaging. The dramatic initial response to corticosteroids further complicated the diagnostic picture. However, steroid responsiveness does not exclude the diagnosis of lymphoma. Corticosteroids induce cytolysis of malignant B cells, and prebiopsy steroid administration can cause rapid tumor involution, a phenomenon described as "ghost tumor." In EBV-associated lymphoproliferative disorders specifically, regression with immunosuppression reduction is well established [13]. Our patient's initial reaction probably reflected direct lymphocytolytic effects rather than the resolution of an autoimmune process. The failure of two bronchoscopic biopsies to establish the diagnosis highlights a major limitation of minimally invasive sampling. The diagnostic yield of endobronchial ultrasound-guided transbronchial needle aspiration (EBUS-TBNA) for lymphoma is approximately 69%, substantially lower than for lung cancer staging [14].

A retrospective comparison found that surgical biopsy achieved diagnosis in a median of eight days with a single procedure, compared to 38 days and up to five procedures in the EBUS-TBNA group [15]. The finding of necrotizing granulomatous inflammation on the second bronchoscopy further misdirected the workup toward infection; however, granulomatous reactions can occur within or adjacent to lymphomas and do not exclude malignancy [16]. Regarding treatment, management of EBV-positive DLBCL arising in immunodeficiency follows PTLD principles: reduction of immunosuppression combined with R-CHOP chemoimmunotherapy [5]. Our patient's end-of-treatment PET/CT showing Deauville score 5 indicates primary refractory disease. For relapsed/refractory DLBCL, CAR T-cell therapy has proved superior event-free survival compared to salvage chemotherapy followed by autologous transplant, with approximately 30% of patients achieving durable remissions [17]. Alternative options include bispecific antibodies such as epcoritamab and glofitamab. This case underscores the need to maintain a broad differential when evaluating pulmonary disease in immunocompromised patients. Tissue diagnosis remains essential when minimally invasive testing is non-diagnostic, and clinicians should consider surgical biopsy early when pulmonary disease is refractory to immunosuppressive therapy in patients at risk for lymphoproliferative disorders.

## Conclusions

This case demonstrates a rare presentation of EBV-positive DLBCL as diffuse pulmonary nodules in a young patient with SLE on chronic immunosuppression. The prolonged diagnostic course highlights the challenge of distinguishing inflammatory, infectious, and malignant causes of pulmonary disease in immunocompromised patients. Initial steroid responsiveness and non-diagnostic bronchoscopic biopsies delayed diagnosis, underscoring the limitations of minimally invasive sampling in suspected pulmonary lymphoma. Ultimately, a surgical lung biopsy was required for definitive diagnosis. Early recognition of lymphoproliferative disorders in patients with autoimmune disease is essential, as timely diagnosis can significantly impact treatment and outcomes.
